# Antibiotic Adsorption by Metal-Organic Framework (UiO-66): A Comprehensive Kinetic, Thermodynamic, and Mechanistic Study

**DOI:** 10.3390/antibiotics9100722

**Published:** 2020-10-21

**Authors:** Mossab K. Alsaedi, Ghada K. Alothman, Mohammed N. Alnajrani, Omar A. Alsager, Sultan A. Alshmimri, Majed A. Alharbi, Majed O. Alawad, Shahad Alhadlaq, Seetah Alharbi

**Affiliations:** 1 Center of Excellence for Nanomaterials for Clean Energy Applications, Joint Centers of Excellence, King Abdulaziz City for Science and Technology, Riyadh 12345, Saudi Arabia; mkalsaedi@kacst.edu.sa (M.K.A.); galothman@kacst.edu.sa (G.K.A.); sshmimri@kacst.edu.sa (S.A.A.); mahsalharbi@kacst.edu.sa (M.A.A.); moalawad@kacst.edu.sa (M.O.A.); shahadibrahim1@gmail.com (S.A.); seatah44@gmail.com (S.A.); 2 National Center for Radioisotopes Technology, Nuclear Science Research Institute, King Abdulaziz City for Science and Technology, Riyadh 12345, Saudi Arabia; mnajrani@kacst.edu.sa

**Keywords:** metal-organic frameworks, antibiotics, water treatment, contaminants removal, recycling

## Abstract

Bacterial antibiotic resistance has been deemed one of the largest modern threats to human health. One of the root causes of antibiotic resistance is the inability of traditional wastewater management techniques, such as filtration and disinfection, to completely eliminate residual antibiotics from domestic and industrial effluents. In this study, we examine the ability of *UiO-66*; a metal-organic framework (MOF); in removing the antibiotic Doxycycline from aqueous environments. This study’s findings suggest that UiO-66 was able to remove nearly 90% of the initial Doxycycline concentration. To correlate the isothermal data, Langmuir and Freundlich models were used. It was determined that the Langmuir model was best suited. Pseudo-first and -second order models were examined for kinetic data, where the pseudo-second order model was best suited—consistent with the maximum theoretical adsorption capacity found by the Langumir model. Thermodynamic analysis was also examined by studying UiO-66 adsorption under different temperatures. Mechanisms of adsorption were also analyzed through measuring adsorption at varying pH levels, thermogravimetric analysis (TGA), Infrared spectroscopy (IR) and Brunauer–Emmet–Teller (BET). This study also explores the possibility of recycling MOFs through exposure to gamma radiation, heat, and heating under low pressure, in order for UiO-66 to be used in multiple, consecutive cycles of Doxycycline removal.

## 1. Introduction

Antibiotics are widely used in human and veterinary pharmaceuticals, agriculture and farming to fight diseases caused by bacteria. However, the administered doses are not completely consumed and about 30–90% of them are released into the environment in their active forms due to runoff effluents. The over-abundant use and improper disposal of antibiotics cause bacteria to develop antibiotic resistance that hinders the ability of antibiotics to effectively cure bacterial diseases. The World Health Organization (WHO) classified antibiotic-resistant bacteria as one of the more prominent threats to public health, food security and economic development [1,2,3,4].

Antibiotics are considered persistent contaminants due to their incessant presence in the ecosystem. Residual antibiotics are continuously detected worldwide in effluents of wastewater [5,6]. Numerous wastewater treatment technologies are employed in order to mitigate antibiotics either partially or completely. Partial elimination methods include disinfection, biological treatment, coagulation, filtration, sedimentation and flocculation. Given the inefficiency of these techniques, oxidation technologies are frequently used to effectively and fully remove antibiotics from wastewater. Such technologies incorporate the generation of ^•^OH radicals to decompose organic pollutants and eliminate them entirely [7,8]. However, in cases where these organic pollutants are significantly present, effective decomposition requires an exceptionally great amount of oxidant, thus forcing industries and municipalities to use oxidation technologies as secondary or tertiary treatments. Other issues associated with oxidation technologies are residual effluent toxicity post-treatment, high costs of application and difficult management of catalysts [9,10].

The aforementioned concerns of oxidation technologies have driven research towards finding alternative and more effective methods of antibiotic elimination. Of these methods are adsorption-based technologies that rely on the porosity of adsorbents to actively and capably remove contaminants from aqueous environments [11,12]. High selectivity, simple operating requirements and low manufacturing cost make these technologies more advantageous than those previously mentioned. Activated carbons, zeolites, multiwalled carbon nanotubes and metal-organic frameworks (MOFs) are examples of highly porous adsorbents that have been proven to successfully remove wastewater contaminants [12,13,14].

MOFs are a relatively novel class of crystalline porous materials comprised of metal ions that are bound together by organic linkers. Due to their versatility, various possible applications of MOFs are currently under way. Gas storage is a heavily researched area, for example—carbon dioxide, methane and other toxic gases have been successfully eliminated in studies using MOFs [15]. Energy transfer and light storage are other examples of MOF application [16]. The exceptionally large surface area, high porosity and tailored tunability of MOFs make them a suitable candidate for adsorption and possibly an even more efficient adsorbent than conventional adsorption materials, such as zeolites and activated carbon for wastewater treatment [15,16,17,18,19].

UiO-66 is a MOF that consists of Zr_6_O_4_(OH)_4_ metal clusters and 1,4-benzenedicarboxylate organic linkers and is one of the most commonly experimented MOFs in aquatic environments due to its hydrophilicity and exceptional adsorption capacity [16,20]. In this study, UiO-66 was used to demonstrate the ability of MOFs to remove small organic contaminants, such as antibiotics, from aqueous media. For this purpose, Doxycycline was used as a model target. 

In this study, it was found that UiO-66 was capable of removing Doxycycline at an almost 90% efficiency in a little less than 30 min. It was also found that the adsorption of Doxycycline onto UiO-66 fits the Langmuir adsorption model and the pseudo-second order kinetics almost linearly. And when the adsorption took place in environments of varying pH values and temperatures to simulate real-life environmental conditions, it was found that higher temperatures were more thermodynamically favorable for adsorption, as well as lower pH values. The possibility of recycling MOFs for multiple consecutive cycles of Doxycycline removal was also examined through three experiments; gamma-irradiation, moderate ambient heating, and moderate heat under low pressure.

## 2. Experimental

### 2.1. Materials and Chemicals

Dimethylformamide (DMF) was purchased from MERCK and used as received. Doxycycline was purchased from Alfa Aesar. Double distilled water (obtained from an in-lab Mill-Q system with a conductivity of 18.2 MΩ.cm) was used to make Doxycycline solutions. Activated carbon and zeolite were purchased from Sigma–Aldrich and were used as received. UiO-66 was synthesized using ZrCl_4_ and terephthalic acid in DMF and hydrochloric, and characterized via Powder XRD and Brunauer–Emmet–Teller (N_2_ isotherms and pore size distribution) according to the renowned procedure in reference number [21]. Briefly, 1: 1.4 molar ratio of ZrCl_4_ (0.54 mmol pre-dissolved in 5:1 *v*/*v* DMF: HCl) to benzene-dicarboxylic acid (pre-dissolved in 10 mL DMF) was heated at 80 °C overnight. The fine white powder was collected through filtration and cleaned with 10% methanol water solution. The content of water was removed by oven drying (100 °C) under vacuum overnight. 

### 2.2. Batch Adsorption Experiments

#### 2.2.1. Adsorption Kinetics

In each batch, for the adsorption experiment for Doxycycline solutions that was conducted, one of the following parameters was changed at a time: solution initial concentration, temperature, and pH. In each experiment, 5 mL of Doxycycline solutions was mixed and stirred (magnetic bar at 400 rpm) with UiO-66 (5 mg). Diluted sodium hydroxide or hydrochloric acid solutions (0.05 M) were used to adjust a solution’s pH. Small aliquots (2–5 μL) of each sample were filtered with a 0.22 μm Millipore syringe-filter before using for Ultraviolet-visible spectroscopy (UV-Vis) measurements. Unknown concentrations were determined by comparing to calibration curves that were obtained from four different concentrations (50, 75, 100, and 150 μM). UV-Vis spectra were recorded before and after adsorption with a NanoDrop One from Thermo Fisher Scientific. The absorption at the wavelength of 275 nm was used to evaluate the reduction in target antibiotic concentration. Adsorption data are the average of 7 experiments, with standard deviation values that do not exceed 6.6%. For comparison purposes, activated carbon and zeolites were also used as adsorbates and their adsorption experiments were conducted as those described above for UiO-66. 

#### 2.2.2. Adsorption Isotherms

Five mL solutions of Doxycycline (at room temperature and pH 7) with different concentrations (250, 500, 750, and 1000 μM) were mixed and constantly stirred (magnetic bar at 400 rpm) with UiO-66 (5 mg). Equilibrium was reached after ~1 h of adsorption and saturation point for UiO-66 was experimentally achieved, where no further reduction in target molecule concentration was observed. Concentrations of Doxycycline solutions were measured as described above. Adsorption capacity of UiO-66 at equilibrium, *Q_e_* (mg/g), and at any given time, *Q_t_* (mg/g), were calculated according to Equation (1) [22] and Equation (2) [17], respectively:

(1) $$Q_{e}=\frac{\left(C_{0}-C_{e}\right)V}{W}$$

(2) $$Q_{t}=\frac{\left(C_{0}-C_{t}\right)V}{W}$$

where *C*_0_ and *C_e_* are the initial and equilibrium concentrations of the Doxycycline solutions (mg/L), respectively; *C_t_* is the concentration of Doxycycline solution at any given time *t*; *V* is the volume of antibiotic solutions (mL); and *W* is the mass of the adsorbent UiO-66 (mg).

### 2.3. Characterization

Thermogravimetric analysis (TGA) was done using a Perkin Elmer TGA7 with a temperature range of 25–800 °C (with a temperature increase rate of 10 °C/min) under constant flow of nitrogen gas and using alumina ceramic crucible. An infrared spectroscope (PERKIN ELMER16F PC FT-IR) equipped with an attenuated total reflectance accessory was used to characterize UiO-66 and UiO-66-Doxycycline complex. Brunauer–Emmet–Teller (BET) measurements were conducted using a Quantachrome Nova touch LX^2^ model. The samples were purged under vacuum for four hours at 120 °C before initiating the analysis. BET surface area was determined via a multi-point method and the pore parameters were calculated via the Dubinin–Astakhov (DA) method.

### 2.4. UiO-66 Recycibility 

For recycling with gamma irradiation: after each adsorption cycle, five mL of Doxycycline solution with UiO-66 (5 mg) was irradiated by gamma ray from Cobalt-60 source in a gamma cell modal 220 from MDS Nordion, Canada. Different irradiation doses (10, 20, and 30 kGy) were achieved by extending the exposure time of the samples to Cobalt-60 source. For recycling with moderate heat: after each adsorption cycle, five mL of Doxycycline solution with UiO-66 (5 mg) was heated in an oven for 24 h at 80 °C. For recycling with moderate heat under vacuum: after each adsorption cycle, five mL of Doxycycline solution with UiO-66 (5 mg) was evaporated in a rotary-evaporation system at elevated temperature and low pressure (75 °C and ~300 torr) for 10 min. After recycling with these methods, UiO-66 was collected and the adsorption of Doxycycline was conducted as detailed above. 

## 3. Results and Discussion 

### 3.1. Percent Removal of Doxycycline by Various Porous Materials

**Figure 1:**
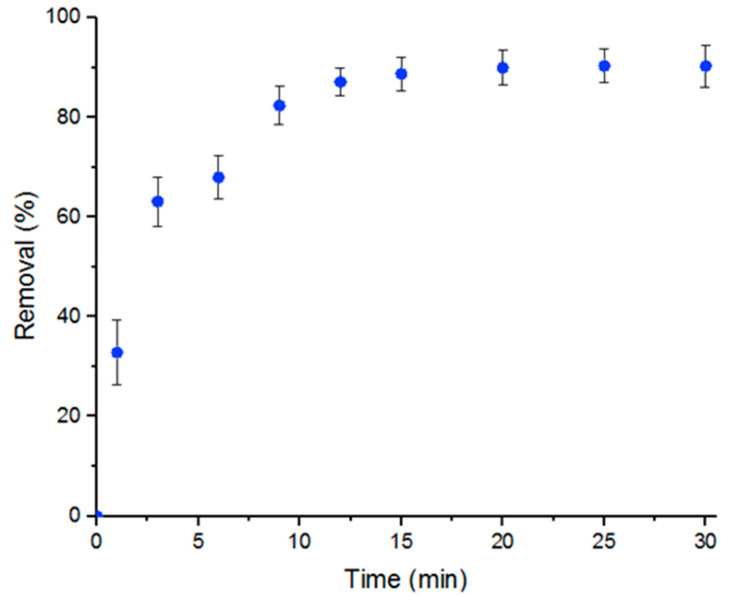
Percent removal of Doxycycline by UiO-66 within 30 min. Error bars represent the standard deviation of three independent adsorption experiments.

The main goal of the study is to demonstrate that MOFs, specifically UiO-66, achieve high efficiency in removing Doxycycline from water. The removal of Doxycycline by UiO-66 was studied in a batch reaction. The percent removal of Doxycycline with respect to time of reaction initiation, which was calculated according to Equation (3), was plotted as shown in Figure 1. Figure 1 shows a maximum percent removal of Doxycycline solution of nearly 100% after 15 min at room temperature and neutral pH conditions. Five mL of a 100-μM solution was used with 5 mg UiO-66.

(3) $$\%R=\frac{A_{o}-A_{t}}{A_{o}}\times 100\%$$

where %*R* is the percent removal of Doxycycline by UiO-66, *A_o_* is the initial absorbance value of the solution prior to adding UiO-66, and *A_t_* is the absorbance of the solution after *t* minutes of adding UiO-66.

**Figure 2:**
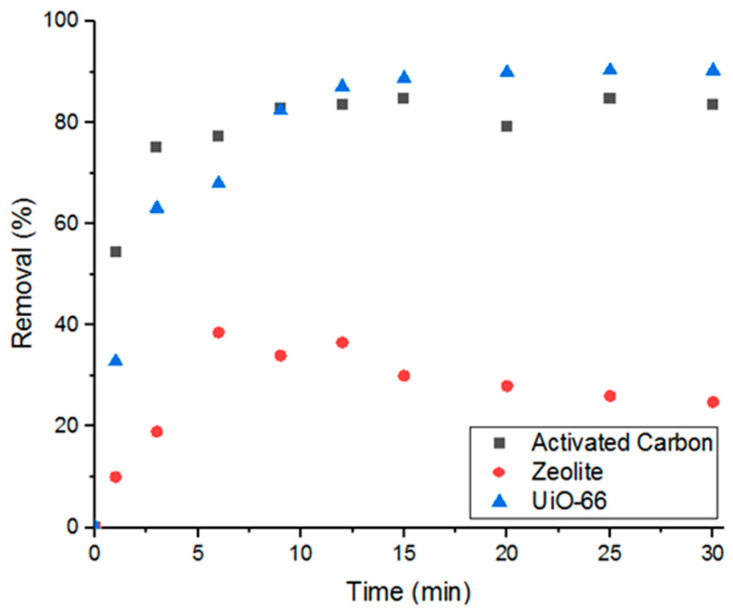
Percent removal of Doxycycline via three different types of porous materials.

Having demonstrated that the MOF is able to successfully adsorb Doxycycline, the work was extended to compare the performance of UiO-66 in removing the target compound to other commonly used porous materials, such as activated carbon and zeolite. As can be seen in Figure 2, UiO-66 showed superior performance to zeolite and comparable performance to activated carbon (when 5 mg of each adsorbent was used). These results demonstrate that UiO-66 possesses great potential to compete with the traditional adsorbents. 

**Figure 3:**
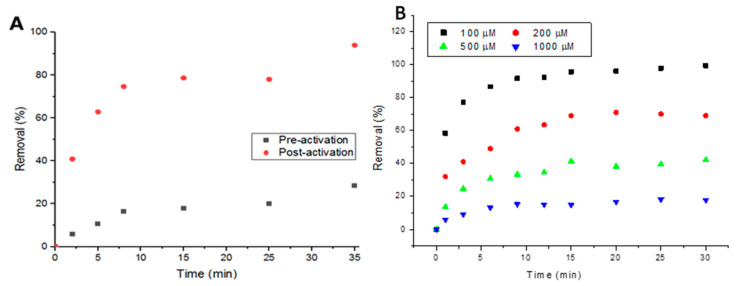
(**A**) Performance of metal-organic framework (MOF) pre- and post-activation with 100-μM solutions of Doxycycline and (**B**) Effect of solution concentration on percent removal.

Activating the metal-organic framework plays a huge role in adsorption, as activation clears up the pores and internal channels from residual starting materials and any other excess molecules. In Figure 3A, it can be seen that the performance of UiO-66 in removing Doxycycline increased from a maximum removal of ~30% to ~90% after activation in 5 mL, 100-μM solutions. To examine the response of UiO-66 to increasing concentrations of Doxycycline, two higher concentrations (500 and 1000 μM) of the target compound were tested. Figure 3B shows that higher removal percentage is associated with lower concentrations and vice versa, confirming that the removal is limited to the MOF’s capacity (thorough details in the following sections).

### 3.2. Adsorption Isotherms

To characterize the adsorbate molecules’ distribution at different equilibrium concentrations in liquid phase, adsorption isotherms are used [7,22]. Furthermore, the nature of interaction between adsorbate molecules and the adsorbent can be better understood through fitting the adsorption data with adsorption models. The adsorption models that were used in this study were the linear-form Langmuir model and Freundlich model, shown in Equations (4) and (5), respectively [7,22]:

(4) $$\frac{C_{e}}{Q_{e}}=\frac{1}{Q_{m}K_{L}}+\frac{C_{e}}{Q_{m}}$$

(5) $$ln\left(Q_{e}\right)=ln\left(K_{f}\right)+\frac{1}{n}ln\left(C_{e}\right)$$

where *C_e_* (mg/L) is the equilibrium concentration of Doxycycline solution, *Q_e_* (mg/g) is the adsorption capacity of UiO-66 at equilibrium, *Q_m_* (mg/g) is the theoretical maximum adsorption capacity of UiO-66, *K_L_* (L/mg) is a Langmuir-adsorption-affinity constant; *K_f_* is a Freundlich empirical constant which corresponds to the relative adsorption capacity of UiO-66; and 1/*n* is a Freundlich-adsorption-intensity constant [22].

**Figure 4:**
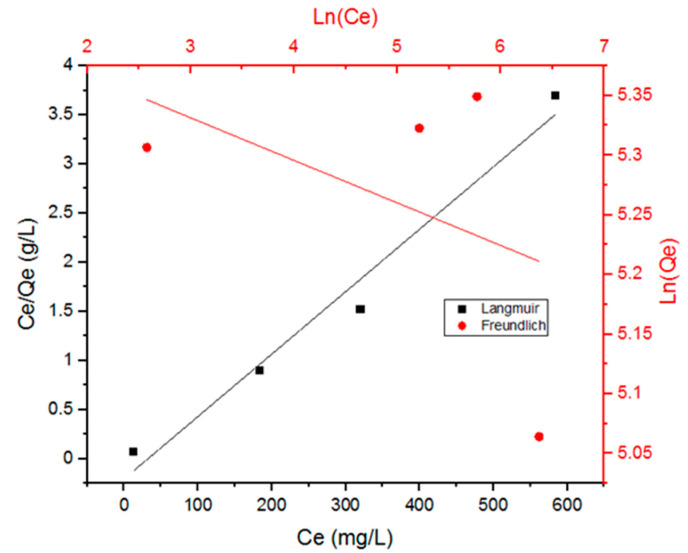
Data fitting with the Langmuir model and the Freundlich model for the adsorption of Doxycycline onto UiO-66.

Figure 4 shows the adsorption data of the Doxycycline into UiO-66 fitted with both models. Table 1 includes the parameters for both models. These two models are known as two-parameter models; they give insight into the adsorption capacity (*Q_e_*) and adsorption-affinity (*K_L_* and *K_f_*) constants. The Langmuir model assumes homogeneous-surface adsorption, meaning that the adsorption sites are of equal accessibility and energy, which results in monolayer adsorption of adsorbate molecules on the surface of the adsorbent saturating the pores and preventing transmigration. The Freundlich model, on the other hand, assumes that the adsorption is non-ideal, irreversible, and occurring through forming multilayers of adsorbate on a non-uniform heterogeneous surface of the adsorbent [7,22].

From Figure 4 and the correlation coefficient values (R^2^) in Table 1, it can be inferred that the Langmuir model seems to be a better fit than the Freundlich model for the adsorption data of Doxycycline into UiO-66 (R^2^ value for Langmuir is close to 0.99). Thus, the adsorption can be characterized as chemisorption (monolayer adsorption), rather than physiosorption (multilayer adsorption) [7]. Monolayer adsorption suggests that the molecules of the adsorbate attach to the surface of the adsorbent and the surface of the adsorbent’s pores by forming one layer of adsorbate molecules next to each other. Given that the dimensions of tetracyclines are 0.86 by 1.27 nm [22] and that the range of pore diameter of UiO-66 is 0.8–1.1 nm [23], Doxycycline is likely to be attaching to the internal surface of the UiO-66’s pores via the shorter side.

The reported *Q_m_* value for the adsorption of Doxycycline onto UiO-66 is comparable to the previously reported value for the adsorption of Doxycycline onto PIM-1 (189 mg/g) [22] and zeolite-hydroxyapatite-activated palm ash (Z-HAP-AA) (186 mg/g) [24] and better than the adsorption of tetracycline (the antibiotic family that Doxycycline belongs to) onto mesoporous silica (44.4 mg/g) [25]. Interestingly, tetracycline adsorption values for activated carbon ranged between 370.04 to 500 mg/g, depending on a rise in temperature [25]. Furthermore, *R_L_*, which is a dimensionless separation factor that expressed the favorable adsorption nature [26] for a Langmuir-model-obeying adsorption process [27], can be calculated as *R_L_* = 1/(1+ *K_L_C*_0_) [28]. The value for *R_L_* indicates an irreversible isotherm (if R_L_ is equal to 0), a favorable isotherm (if *R_L_* is between 0 and 1), a linear isotherm (is R_L_ is equal to 1), or an unfavorable isotherm (if *R_L_* is bigger than 1). Using *K_L_* from Table 1, *R_L_* was calculated to be 0.244, which is between 0 and 1, meaning that the adsorption isotherm is favorable [7,26,27].

### 3.3. Adsorption Kinetics

**Figure 5:**
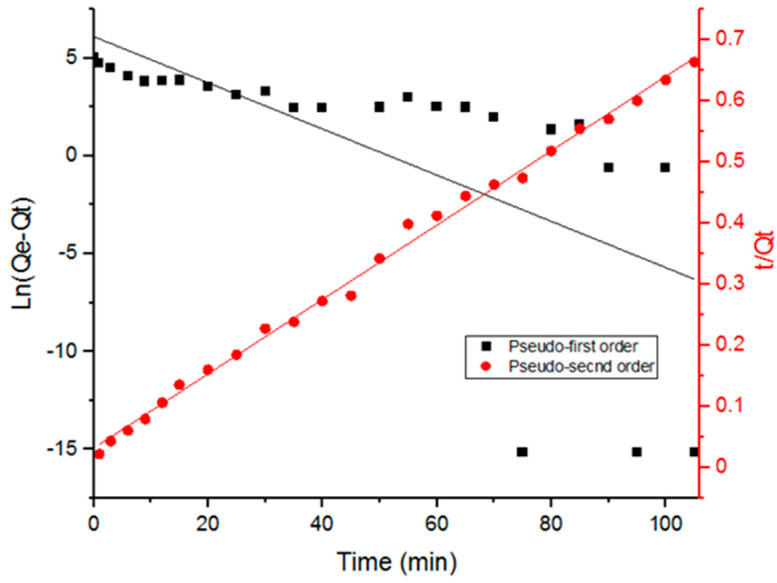
Fitting data with pseudo-first and pseudo-second order kinetics of Doxycycline adsorption onto UiO-66.

Adsorption kinetics data were analyzed (Figure 5) using two kinetic models: pseudo-first order and pseudo-second order in their linear forms as shown in Equations (6) and (7) [7,27,29]:

(6) $$\ln\left(Q_{e}-Q_{t}\right)=\ln\left(Q_{e}\right)-K_{1}t$$

(7) $$\frac{t}{Q_{t}}=\frac{1}{{K_{2}Q_{e}}^{2}}+\frac{t}{Q_{e}}$$

where *Q_e_* and *Q_t_* are the adsorption capacity of UiO-66 at the equilibrium and at any given time *t*, respectively; *K*_1_ (min^−1^) and *K*_2_ (g/mg min) are the moduli of pseudo-first order and pseudo-second order adsorption, respectively [7,27,29].

Table 2 includes the parameters obtained from fitting the data with the adsorption kinetic models. It is evident in Figure 5 that adsorption data fit the pseudo-second order kinetics better. Furthermore, the correlation coefficient values in Table 2 (R^2^) indicate a better fit with the pseudo-second order model. The pseudo-second order kinetic model suggests that chemical adsorption is the dominant mechanism, involving electrostatic attraction. The interaction between the different functional groups that Doxycycline possesses and UiO-66 has within its cage-like structure can explain the dominance of chemical adsorption. Doxycycline, as well as the organic ligands in UiO-66, are abundant with hydroxyl groups and carbonyl groups; furthermore, Doxycycline has amine groups. The presence of such functional groups could be the cause for intermolecular forces, such as hydrogen bonds, that explain the dominance of chemosorption.

Additionally, the adsorption capacity at equilibrium predicted by the pseudo-second-order model from Table 2 (164 mg/g) is consistent with the trend of the maximum theoretical adsorption capacity found by Langumir model in Table 1 (156.25 mg/g).

### 3.4. BET surface area, IR and TGA Characterizations of the Adsorption of Doxycycline onto UiO-66

**Figure 6:**
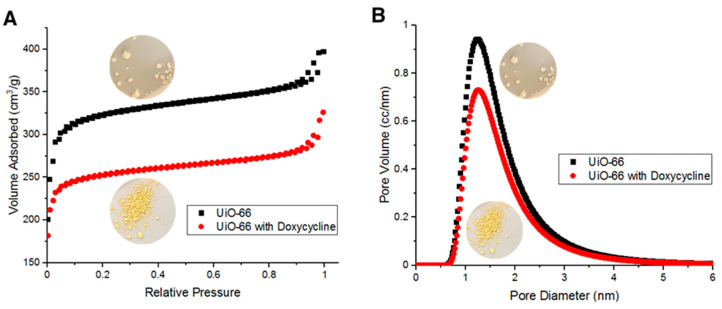
(**A**) Brunauer–Emmet–Teller (BET) adsorption/desorption isotherms for fresh UiO-66 and Doxycycline-filled UiO-66 and (**B**) pore diameter distribution with respect to volume of fresh UiO-66 and Doxycycline-filled UiO-66.

BET surface area measurements indicate that fresh UiO-66’s surface area is about 856 m^2^/g. Such large surface area is due to the adsorption of N_2_ molecules both on the surface of UiO-66 and within its pores. Figure 6A shows UiO-66 and Doxycycline-filled UiO-66 BET adsorption/desorption isotherms. Figure 6B shows the distribution of pore diameter of UiO-66 and Doxycycline-filled UiO-66 with respect to sample volume. The adsorption/desorption curves in Figure 6A are type II isotherms (as classified by IUPAC). Hysteresis loops were observed upon N_2_ desorption at high relative pressure, which are due to capillary condensation then evaporation. Figure 6B indicates that UiO-66 is microporous in majority (diameter < 2 nm) with a fraction of its volume being mesoporous (diameter > 2 nm). Upon the adsorption of Doxycycline, the UiO-66 surface area dropped to 669 m^2^/g, which is a 187-m^2^/g decrease in surface area (~27.9% decrease in surface area). Such a reduction in surface area is comparable and relatively higher than other reported porous adsorbents of Doxycycline. For example, PIM-1 was reported to have 60 m^2^/g reduction in surface area upon the adsorption of Doxycycline [7]. The adsorption/desorption isotherms in Figure 6A and the pore diameter distribution in Figure 6B both show a consistent behavior of the overall data for fresh UiO-66 adsorbing more volume and having more volume with respect to each pore diameter than the overall data for UiO-66 after adsorbing Doxycycline. This pattern is due to the effect that the adsorption of Doxycycline has on the internal free volume of UiO-66 (reduction in internal free volume).

**Figure 7:**
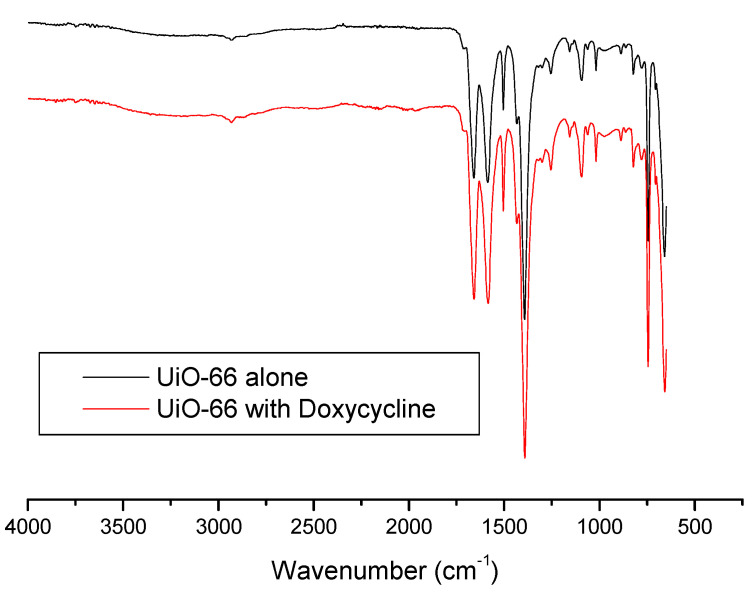
FTIR spectra for fresh UiO-66 and Doxycycline-filled UiO-66.

IR spectroscopy was also used to get further insight and understand the adsorption process more. Figure 7 shows IR spectra of UiO-66 and Doxycycline-filled UiO-66. The sharp, medium-intensity peak around 1650 cm^−1^ corresponds to the alkene double bond (C=C) in the structure of both UiO-66 and Doxycycline. The relatively weak peaks at ~1750 cm^−1^ correspond to carbonyl groups (C=O) in both structures. Is it important to note that peaks for hydroxides (O-H) and amines (N-H) found in Doxycycline are not present, which are strong/broad and medium peaks at around 3400–3700 and 3300–3350 cm^−1^, respectively. This could indicate either of the following or both: the shielding effect that porous nature of UiO-66’s structure has on the adsorbed Doxycycline within its pores, and/or the weak signaling if Doxycycline molecules in response to IR. A similar behavior has been reported with the adsorption of Doxycycline onto PIM-1 [7]. It is important to note, however, that this specific characterization study does not give much insight on the adsorption process, as both spectra seem to be almost identical.

**Figure 8:**
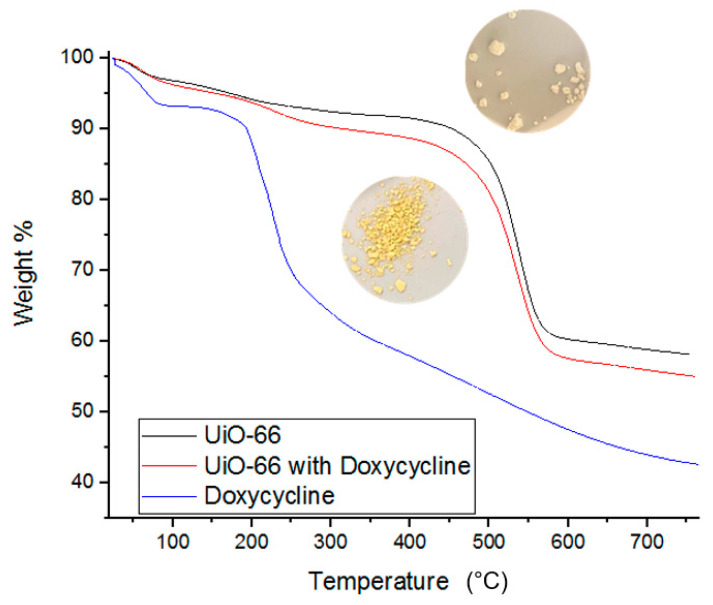
TGA data for fresh UiO-66, fresh Doxycycline, and Doxycycline-filled UiO-66. Images of Doxycycline loaded (bottom) and unloaded UiO-66 (top) are provided.

BET and IR spectroscopy data are supported by TGA measurements. TGA measurements provide insights into the thermal decomposition behavior of each component of the adsorption system before and after interaction. TGA data are conventionally presented as temperature increase versus % weight loss [7]. Figure 8 shows that the thermal decomposition of pure Doxycycline powder (blue curve) begins its sharp weight loss at 200 °C. Figure 8 also shows the decomposition of UiO-66 alone and when loaded with doxycycline. UiO-66 alone (image of white powder and black curve in the figure) showed thermal resistance up to approximately 400 °C, after which notable weight loss is observed. On the other hand, the thermal decomposition of UiO-66 with Doxycycline (image of yellow powder and red curve in the figure) showed about an additional 5% increase in weight loss compared to UiO-66 alone, and the additional 5% loss in weight occurs gradually between 200 and 325 °C. Such difference in thermal behavior of loaded and unloaded UiO-66 with doxycycline is evidence of the successful adsorption of doxycycline by UiO-66. Similar TGA data were reported for the adsorption and thermal decomposition of antibiotics, including Doxycycline, which adsorbed onto polymer of intrinsic micro-porosity (PIM-1) [7].

### 3.5. Effect of Experimental Conditions on the Adsorption of Doxycycline 

#### 3.5.1. Solution pH 

**Figure 9:**
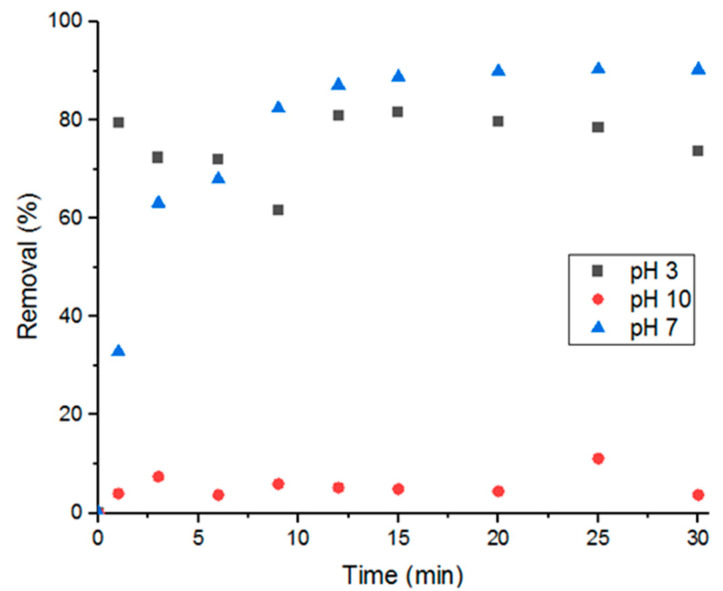
Effect of solution pH on percent removal.

Three different solutions each with a pH of 3, 7, or 10 were tested for adsorption at room temperature (5 mL of 100-uM solutions). Figure 9 shows that basic solutions have the least adsorption performance, acidic solutions have the fastest adsorption, and neutral solutions have the highest removal percent. Difference in pH affects the charges on both the adsorbate and adsorbent, which then affects molecular adsorption. Acidic environments promote deprotonation, making Doxycycline’s functional groups (amines, carbonyls, and hydroxide groups) more negatively charged. And basic environments would promote the opposite; less-negatively-charged Doxycycline molecules. That being said, the high adsorption efficiency demonstrated in pH 3 solutions compared to pH 10 could hint to higher degree of electrostatic repulsion in basic conditions, which leads to lower adsorption. This also could hint to UiO-66 surface being dominated by positive charges [7,27,29].

#### 3.5.2. Effect of Temperature and Thermodynamic Parameters on Adsorption of Doxycycline 

**Figure 10:**
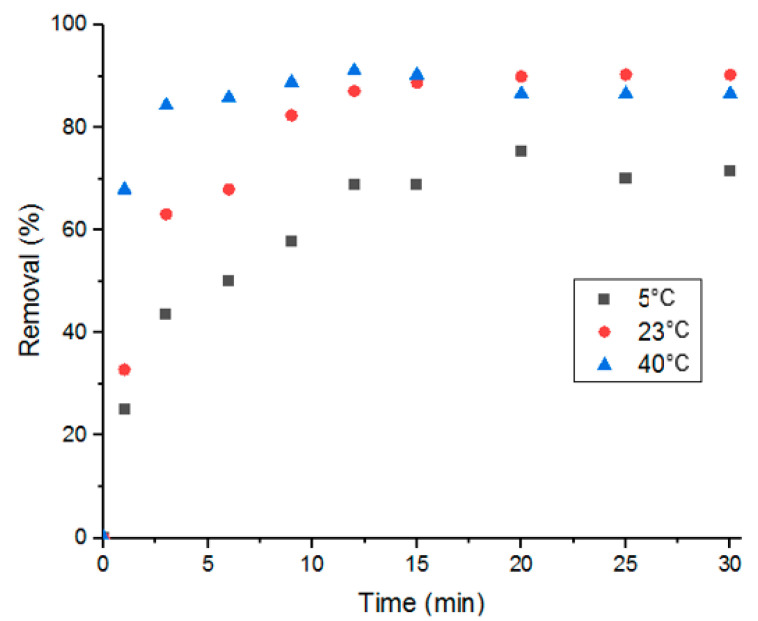
Percent removal of Doxycycline via UiO-66 at different solution temperatures.

In this study, influence of the adsorption process’s temperature and determination of thermodynamic parameters were conducted at three different temperatures: 5, 25 and 40 °C. As shown in Figure 10, adsorption performance was better with solutions at room temperature and at 40 °C. And when comparing adsorption at room temperature to adsorption at 40 °C, it is evident that the higher the temperature, the faster the adsorption is. This stems from the effect that heat has on diffusion; elevated temperatures promote faster molecular diffusion.

Gibbs free energy (Δ*G*^0^), enthalpy (Δ*H*^0^) and entropy (Δ*S*^0^) of the adsorption of Doxycycline onto UiO-66, were determined by *K_d_*, which is the variation of solute distribution coefficient between the solid and liquid phases, via Equations (8)–(10) [7,27,30]:

(8) $$K_{d}=\frac{q_{e}}{C_{e}}$$

(9) $$∆G^{0}=-RT\;Ln\left(1000\times K_{d}\right)$$

(10) $$Ln\left(1000\times K_{d}\right)=\frac{-∆H^{0}}{RT}+\frac{∆S^{0}}{R}$$

where *R* is the universal gas constant (8.314 J/(mol K)), *T* is the absolute temperature of the system in Kelvin, and *K_d_* is multiplied by 1000 to become dimensionless [7,31,32,33]. *K_d_* must be dimensionless for the unit of Δ*G*^0^ to become J/mol in Equation (8), as the gas constant and temperature are J/(mol K) and K, respectively. Because the adsorption of Doxycycline was investigated in aqueous solutions with low concentrations of the antibiotic, the dimensionality of *K_d_* (L/g) can be made dimensionless by multiplying the distribution coefficient by 1000, since 1 L = 1000 g, and the solution density is 1 g/mL [7,31,32,33]. Δ*H*^0^ and Δ*S*^0^ were calculated from the slope and the y-intercept, respectively, of the line from the van’t Hoff equation (*Ln*(1000 × *K_d_*) versus 1/T). The calculated Δ*G*^0^ values were all negative (shown in Table 3), which are indicative of spontaneous adsorption [31,34]. Spontaneity, which can be thought of as the increase in the value of the absolute value of the Δ*G*^0^ values, increased as the temperature increased, which also points at the previous statement that higher temperatures promote adsorption via faster diffusion.

Using of the dimensionless *K_d_* to calculate Δ*H*^0^ and Δ*S*^0^ might not be the most appropriate for the Doxycycline-UiO-66 adsorption system, as the van’t Hoff linear fit resulted in an R^2^ value of 0.58. Previous studies showed that such method is sometimes not the best, such as in the adsorption of different antibiotics onto PIM-1 [7], and the adsorption of cadmium onto orange peel [31].

### 3.6. Recycling UiO-66 for Adsorption Application

**Figure 11:**
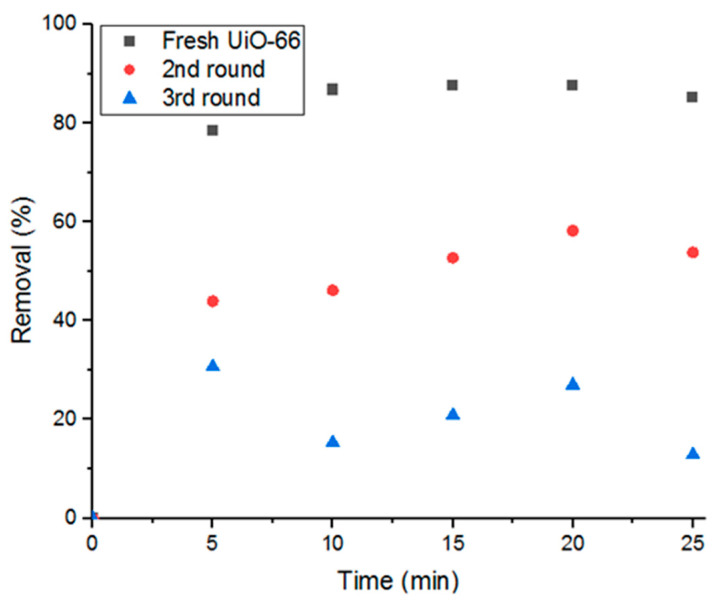
Decrease of removal percent with cyclic usage of adsorbent.

Reusing the adsorbent in consecutive adsorption cycles is another aspect of this study. As shown in Figure 11, cyclic usage of UiO-66 reduces the adsorption performance by 37.5% removal of Doxycycline in each cycle until nearly full saturation is reached after the third cycle. Therefore, the present study investigates three main recycling methods in order to regain its high removal efficiency and purge the material from the previously adsorbed Doxycycline. The methods investigated are gamma-irradiation, moderate ambient heating, and moderate heat under low pressure.

The use of gamma-radiations is thought to form the highly oxidizing species ^•^OH radicals via water radiolysis, which is a well-known advanced oxidation process [9,10]. In this study, ionizing gamma irradiation from Co-60 source (a radioactive source) was used to initiate the production of various short-lived water radiolysis species according to Equation (11) [9,10]:

H_2_O ^γ-rays^ (2.6) e^−^_aq_ + (0.6) H^•^ + (2.7) OH^•^ + (0.7) H_2_O_2_ + (2.7) H_3_O^+^ + (0.45) H_2_ (11)

where the numbers in the brackets represent the amount of produced radicals per 100 eV energy [9,10]. The highly oxidizing hydroxyl radical (^•^OH) and reducing hydrated electrons (e^−^_aq_) are produced with relatively high yield compared to the other short-lived species presented in Equation (11). Hydrated electrons and hydrogen radicals (H^•^) undergo subsequent reaction with the dissolved oxygen to produce inactive species (including superoxide radical anion and hydroperoxyl radical) [9,10]. H_2_O_2_ and H_2_ are produced with low yield and known to have limited chemical reactivity. Thus, hydroxyl radicals are the only oxidizing species that are produced in high yield and with high chemical reactivity during water radiolysis process by ionizing irradiation [9,10].

**Figure 12:**
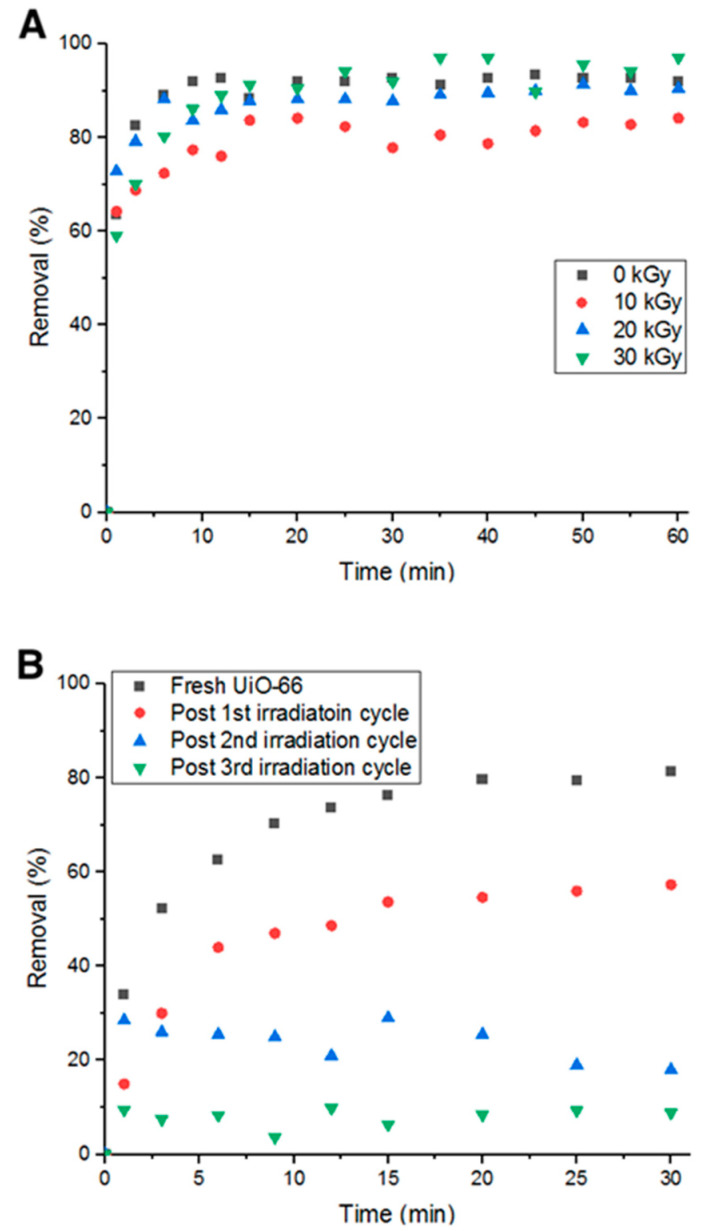
(**A**) Percent removal of Doxycycline with irradiated MOF (different gamma-ray dosages) and (**B**) Percent removal with irradiation cycles.

It was hypothesized that hydroxyl radicals would break the bonds between the adsorbent and adsorbate, leading to more vacant pore volume and surface area [11]. However, prior to utilizing this method, irradiation with 10, 20, and 30 KGy were tested on UiO-66 to ensure the exposure to irradiation does not affect its adsorption capability, which would then affect the removal efficiency. Figure 12A shows the percent removal of Doxycycline by fresh UiO-66 and UiO-66 that has been exposed to 10, 20 and 30 KGy. It is evident from the figure that the adsorption performance did not significantly change.

Fixing the radiation intensity at 10 KGy, four consecutive cycles of adsorption were run with four hours of irradiation as a recycling step between each cycle. In Figure 12B, it can be inferred that the average drop in removal percent decreased to 23.7% compared to the previous 37.5% (non-recycled). This is attributed to the irradiation’s ability to break bonds between Doxycycline and UiO-66 via the formation of ^•^OH radicals from the aqueous solution.

**Figure 13:**
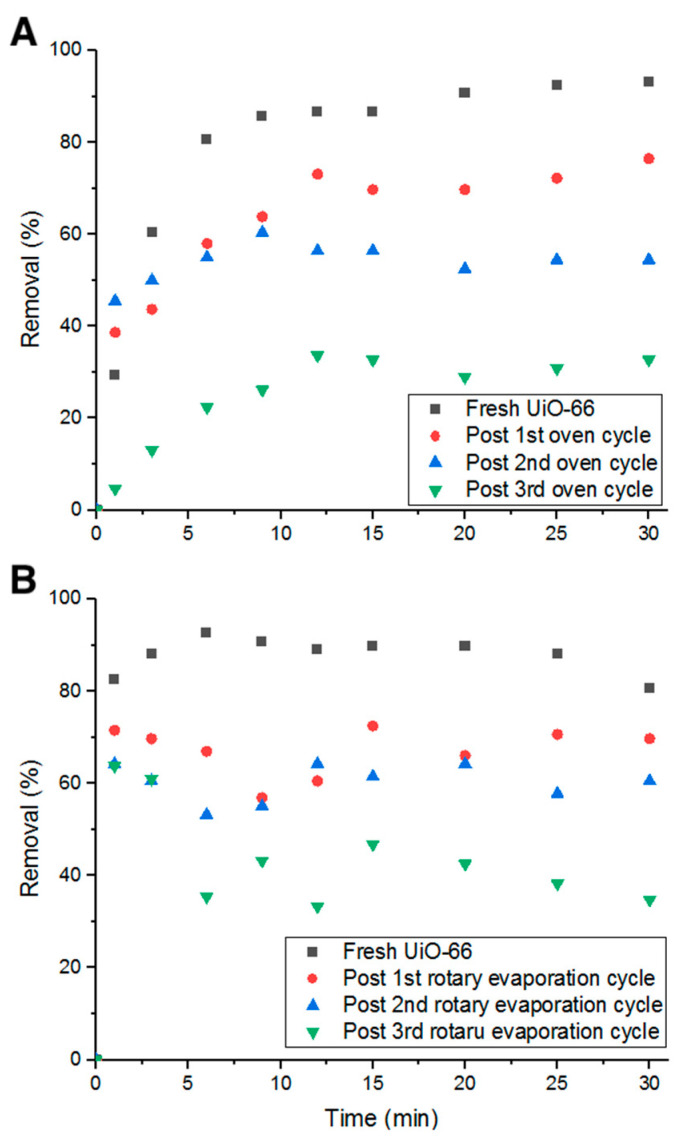
Percent removal with (**A**) oven heating cycles and (**B**) rotary evaporation cycles.

Heating in an oven for 24 h at 80 °C between each adsorption cycle was investigated. As inferred from Figure 13A, the decrease in percent removal dropped to 20%. This could be due to the effect heat has on Doxycycline. As heat decomposes the antibiotic (confirmed by UV-Vis measurements), pores are freed up for the cycles to follow. Additionally, after 24 h of heat, water content is completely eliminated, which could play a role in the removal and detachment of Doxycycline from the structure of UiO-66 due to the absence of electrostatic forces with the evaporation of water molecules from the adsorption medium.

The third recycling method investigated is moderate heat (evaporation) under vacuum for 10 min at elevated temperature (75 °C and ~300 torr). With this recycling method, the average decrease in percent removal of Doxycycline dropped to 9.61%, which is lower than all previous recycling methods (shown in Figure 13B). Such a low decrease in removal efficiency can be attributed to the combination of chemical decomposition of Doxycycline by elevated temperatures, elimination of the electrostatic forces and other chemical bonds that keep Doxycycline molecules bound to UiO-66’s structure, and physical dissociation of Doxycycline from UiO-66 by low-pressure force. This method is more advantageous than the previously tested methods as it recycles more efficiently in less time. Although all examined methods could not fully reactivate the adsorption property of UiO-66, they are still more economically and environmentally beneficial to consider for recycling adsorbents, even partially, to minimize treatment cost and waste production.

### 3.7. Potential Large Scale Deployment of MOF in Water Treatment

MOFs are novel class of crystalline porous materials comprised of metal ions that are bound together by organic linkers. The key cost aspects of synthesizing MOFs are the prices of linkers, metal clusters, and solvents. It was estimated that the baseline costs for synthesizing a number of MOFs (including UiO-66) range from $35 to $71/kg when solvothermal synthesis route is used [35]. The cost of synthesis can be significantly reduced by 34% to 83% when other synthetic routes are utilized, such as liquid assisted grinding and aqueous synthesis [35]. By carefully recycling solvents, unreacted metal salts, and unreacted linkers; a significant reduction in the cost of MOF production to achieve $10/kg was estimated [35]. Furthermore, the implementation of adsorption technology in water treatment plants as secondary or tertiary treatment strategy is an already mature practice [36]. Large-scale adsorption units with particular engineering designs are available for the demonstration of an adsorbent of interest [36]. Thus, MOFs can be incorporated into these treatment stages. Used MOFs can be recycled using methods explored in the present study, or other alternative recycling methods currently being explored.

## 4. Conclusions

Experimental data show promising results for the future of MOFs in wastewater treatment of antibiotics. The study proves that UiO-66 is able to remove up to 90% of the initial concentration of Doxycycline in an aqueous environment under optimized conditions. The isothermal data presented were consistent with Langmuir modeling. The pseudo-second order model best describes the kinetic data. The study shows that MOFs are comparable to other porous materials for antibiotic adsorption. The possibility of recycling MOFs has also been investigated through exposure to gamma irradiation, heating and heat under vacuum. Findings suggest that, although unable to fully reactivate MOFs full potential, these methods are encouraging probabilities of a cost-effective and environmentally cautious future for MOFs in wastewater management. Further investigation into recycling methods is required, as well as investigations into avenues of scaling up and industrializing MOFs for wastewater treatment of antibiotics and possibly other harmful water contaminants.

## Figures and Tables

**Table 1:** Adsorption parameters for the Langmuir and Freundlich adsorption models.

Model	*K_L_* or *K_f_*	*Q_m_* (mg/g) or 1/*n*	R^2^
Langmuir	0.031053	156.25	0.98
Freundlich	230.12	0.0357	0.20

**Table 2:** Adsorption kinetics parameters for pseudo-first and pseudo-second orders of Doxycyline adsorption onto UiO-66.

Adsorption Order Model	*K*_1_ (Min^−1^) Or *K*_2_ (G/Mg Min)	*Q_e_* (Mg/G)	R^2^
Pseudo-first order	0.118	433	0.44
Pseudo-second order	0.00116	164	0.99

**Table 3:** *K_d_* and Δ*G*^0^ values for adsorption of Doxycycline onto UiO-66 at different temperatures.

Temperature (K)	*K_d_* (Dimensionless)	Δ*G*^0^ (kJ/mol)
278	2208	−17.8
296	8952	−22.4
313	6445	−22.8
